# Short-Term Outcomes of a Structured Self-Rehabilitation Program After Mini-Open Latarjet Procedure in Military Personnel: A Prospective Observational Study

**DOI:** 10.3390/medsci13040307

**Published:** 2025-12-07

**Authors:** Kyriakos Bekas, Ioannis Bampis, Alexandros Stamatopoulos, Apostolos-Apollon Papadimitriou, Konstantinos Vamvakeros, Ioannis Kechagias, Achilleas Boutsiadis

**Affiliations:** 1 Orthopaedics Department, 401 General Army Hospital of Athens, 115 25 Athens, Greeceboutsia@gmail.com (A.B.); 2 Bioclinic Advanced Shoulder Service, 115 24 Athens, Greece; 3 Great Western Hospitals NHS Foundation Trust, Swindon SN3 6BB, UK

**Keywords:** shoulder surgery, COVID-19, self-rehabilitation, anterior shoulder instability, mini-open Latarjet

## Abstract

**Background/Objectives:** The COVID-19 pandemic limited access to in-person physiotherapy, raising concerns about post-operative rehabilitation outcomes. This prospective observational study, without a control group, evaluated whether a self-rehabilitation protocol following a mini-open Learjet procedure influenced short-term clinical outcomes in active military personnel. **Materials and Methods:** We prospectively enrolled 18 patients (19 shoulders) undergoing mini-open Latarjet between May and October 2020. Patients performed a standardized self-rehabilitation protocol starting on the first post-operative day, with progressive range-of-motion (ROM) exercises added at two weeks. Pain was assessed using the Visual Analog Scale (VAS), ROM was recorded at each follow-up, complications were noted, and patient satisfaction was evaluated at 12 weeks. **Results:** A total of eighteen patients were prospectively enrolled in the study. At 12 weeks, mean VAS decreased from 1.2 ± 0.6 at week 1 to 0 at week 4 onward. The mean drug consumption was 2.5 ± 0.7 tablets/day only for the first week. Mean assisted forward flexion improved from 155° ± 10° at week 1 to 180° in all patients by week 4. External rotation reached 60° ± 5°at 4 weeks, 75° ± 4° at 8 weeks, and 80° ± 3°at 12 weeks, with no deficits compared to the contralateral side. Internal rotation improved to the T7 level by week 8 and remained stable in week 12. No complications, recurrent instability, or graft displacements were reported. Patient satisfaction at 12 weeks was assessed using a 0–10 numeric rating scale, with a mean score of 9.5 ± 0.4. **Conclusions:** Implementation of a self-rehabilitation protocol after mini-open Latarjet surgery was associated with favorable short-term outcomes in young military patients, including early recovery, high satisfaction, and absence of complications. Further validation of these findings will require larger, rigorously controlled studies.

## 1. Introduction

Anterior shoulder instability is a common condition in young, active populations, particularly among athletes and active-duty military personnel [1]. The Latarjet procedure is a surgical mini-open technique used to treat traumatic anterior shoulder instability. Even though the specific indications differ amongst surgeons, patients with anterior glenohumeral instability that are unlikely to have a successful outcome from either an arthroscopic or open anatomical Bankart repair, patients with glenoid bone loss > 10% (measured in computed tomography) and ones with Hill–Sachs lesions that engage glenoid defect (off-track) are more likely to benefit from this procedure [2]. In addition, patients who underwent the Latarjet procedure return to sports earlier [3,4,5] and have lower rates of recurrent instability [3,6,7,8,9] in comparison to those who underwent Bankart repairs.

Post-operative rehabilitation is a crucial determinant of functional recovery and long-term outcomes. According to the literature, the rehabilitation program after the Latarjet procedure includes a period of 2–3 weeks of immobilization in a shoulder brace, followed by supervised physiotherapy to restore range of motion, strength, and shoulder stability for additional 3–6 weeks [10,11]. The adverse effects of delayed mobilization after shoulder surgery have been highlighted in the recent literature [12,13,14]. Rehabilitation typically involves a combination of exercises and therapy to help the individual regain strength, flexibility, and range of motion in the affected shoulder. This includes exercises to improve shoulder mobility and stability, as well as activities to strengthen the shoulder and upper arm muscles.

During the COVID-19 pandemic, in-person physiotherapy was severely restricted in Greece. Rehabilitation programs were adapted to protect patients and staff. Clinicians and patients used alternative strategies. In this context, self-directed or home-based programs, with remote follow-up became practical. Several studies report that tele-rehabilitation and self-managed recovery yield early outcomes similar to supervised physiotherapy after orthopedic procedures, including shoulder surgery. Objective indicators for recovery after shoulder stabilization include range-of-motion symmetry, inter-limb strength balance, and functional mobility [3,15,16,17,18,19]. Structured self-rehabilitation protocols included pre-screening for symptoms, use of personal protective equipment and social distancing. Virtual or tele-rehabilitation services were also offered. Evidence remains limited, especially for populations with high physical demands such as military personnel.

This study evaluates short-term outcomes of military patients with anterior shoulder instability treated by mini-open Latarjet and proposes an immediate self-rehabilitation protocol with regular video consultations during COVID-19. We hypothesized that this approach does not adversely affect the range of motion, pain, early functional recovery, or satisfaction.

## 2. Materials and Methods

### 2.1. Study Design and Ethical Approval

This prospective observational study was conducted at the Orthopaedics Department of the 401 General Army Hospital of Athens, May–October 2020.

Ethical approval and written informed consent were obtained prospectively prior to patient enrollment in 2020. Due to the institute’s regulations and post-pandemic restructuring, the hospital’s Ethics Committee approved the study in September 2024 (Approval No. 2024-15-9, Date: 15 September 2024). The updated approval number re-issued during institutional digital restructuring in 2024 reflects document archival updates rather than the timing of initial ethical over-sight. All patients gave written informed consent before surgery.

### 2.2. Patient Selection

Eighteen male military patients with recurrent traumatic anterior shoulder instability were included. One patient underwent bilateral surgery, resulting in 19 operated shoulders. Each shoulder was analyzed independently because surgery and rehabilitation were performed separately: one patient contributed two observations. Pre-operative planning included X-rays (anterior–posterior [AP], scapular Y-View, and Bernageau views) and computed tomography (CT) scans to evaluate glenoid bone loss. Inclusion criteria were traumatic anterior shoulder instability, glenoid bone loss >10% (by CT), Hill–Sachs lesions engaging the glenoid defect (off-track, referring to a lesion that interacts with the glenoid rim), age under 40, and physically active service members. Exclusion criteria were previous surgery on the same (ipsilateral) shoulder, neurological deficits, instability unrelated to trauma or affecting multiple directions (multidirectional), and other conditions affecting rehabilitation compliance.

### 2.3. Surgical Technique

All procedures were performed by the same experienced surgeon (A.B.) using a mini-open Latarjet technique under general anesthesia and beach-chair positioning. A 4–5 cm incision (deltopectoral approach) was made vertically from the tip of the coracoid toward the axillary fold. The coracoacromial ligament was exposed and incised 1 cm from the coracoid attachment. The coracohumeral ligament and the pectoralis minor were released. Coracoid osteotomy was performed at the junction of the horizontal and vertical components. A parallel drill guide was used to improve the placement of the coracoid graft [20]. Then, the coracoid process was fixed with two 3.75 mm full-threaded screws with a lag-screw way of positioning [21]. Immediately postoperatively, a shoulder brace in abduction and neutral rotation was placed in all cases.

### 2.4. Self-Rehabilitation Protocol

Due to COVID-19 restrictions, patients followed a structured self-rehabilitation program instead of formal physiotherapy. The protocol comprised three phases. In Phase 1 (from the first post-operative day for 2 weeks), we encouraged the patients to perform active-assisted forward flexion (AFF) exercises in 5 cycles, repeating them 5 times per day within pain-free limits [3]. The patients should also perform pendulum exercises three times daily. Hand, wrist, and elbow mobility was encouraged without restriction.

Phase 2 (Weeks 2–4): patients added gentle active-assisted external rotation (ER) exercises to the pain-free limit. Internal rotation (IR) exercises were also performed with the arm at the side. Scapular setting and retraction exercises were initiated as well.

In Phase 3 (fourth post-operative week onwards), active ROM exercises in all plans were encouraged within pain-free limits. Closed-chain stabilization exercises were added (e.g., wall slides, table weight shifts). Progressive strengthening with elastic bands and stretching exercises were also added, to maintain full range of motion. By the end of phase 3 the patients were encouraged to gradually return to daily activities and military physical tasks as tolerated.

All the patients received written instructions with illustrations and underwent virtual video consultations in weeks 1, 2, 4, 8, and 12 to verify technique and adjust progression.

### 2.5. Clinical Evaluation

Pain was assessed using a Visual Analog Scale (VAS). Range of motion (ROM) was measured for active forward flexion (AFF), external rotation (ER), and internal rotation (IR) at each follow-up. ROM measurements were performed using a standard handheld goniometer by the same surgeon (A.B.) to ensure consistency. Postoperative analgesia consisted of Lonalgal^®^ (paracetamol 500 mg + codeine 30 mg), with a mean intake of 2.5 ± 0.7 tablets/day during the first postoperative week. Analgesic consumption, complications, and patient satisfaction (0–10 scale) were recorded at 1, 2, 4, 8, and 12 weeks postoperatively.

Twelve weeks post-operatively, the position and the healing process of the graft were evaluated by radiographic evaluation (True AP, Bernageau, Y-view).

### 2.6. Statistical Analysis

Given the small sample size and the descriptive nature of this pilot study, inferential statistical testing (e.g., repeated-measures ANOVA) was not performed because it would not provide meaningful power or reliability. Instead, trends were analyzed descriptively to illustrate postoperative changes over time. Future larger studies will be needed to support formal hypothesis testing. Continuous variables were expressed as mean ± standard deviation (SD). Categorical variables were summarized as counts and percentages.

## 3. Results

A total of 18 male military patients (19 operated shoulders) were included in the study. The mean age was 28 ± 7 years. The dominant arm was affected in 12 cases (63%), and the right shoulder was more commonly involved (14 shoulders, 74%). All patients completed the 12-week follow-up period, and none were lost to follow-up. Table 1 summarizes the main outcomes.

### 3.1. Pain and Analgesic Use

Mean postoperative pain, as measured by the VAS score, decreased steadily over time. The mean VAS was 1.2 ± 0.6 at week 1, 0.8 ± 0.5 at week 2, and 0 from week 4 onward. Patients used Lonalgal^®^ (Boehringer Ingelheim International GmbH, Athens, Greece, paracetamol 500 mg + codeine 30 mg), with a mean intake of 2.5 ± 0.7 tablets/day during the first postoperative week only. No patients required pain medication beyond the initial week.

### 3.2. Range of Motion (ROM)

**Figure 1:**
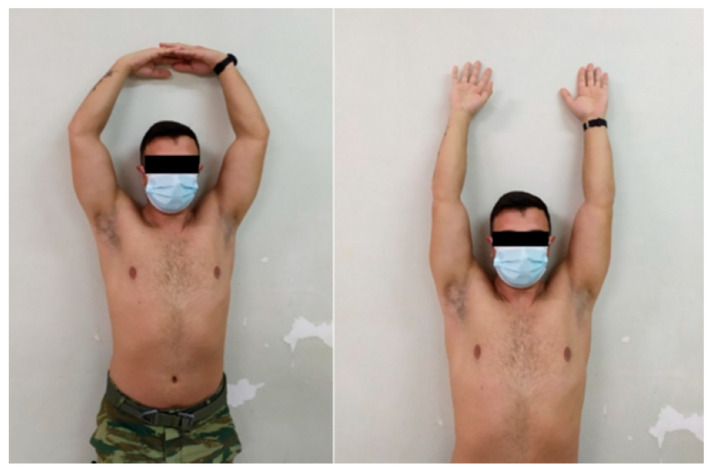
Active forward flexion 4 weeks postoperatively.

All patients achieved progressive and full restoration of shoulder motion by week 12. The mean assisted AFF was 155° ± 10° and 170° ± 5° at 1 and 2 weeks, respectively, and during the following time intervals it reached 180° in all patients (Figure 1).

**Figure 2:**
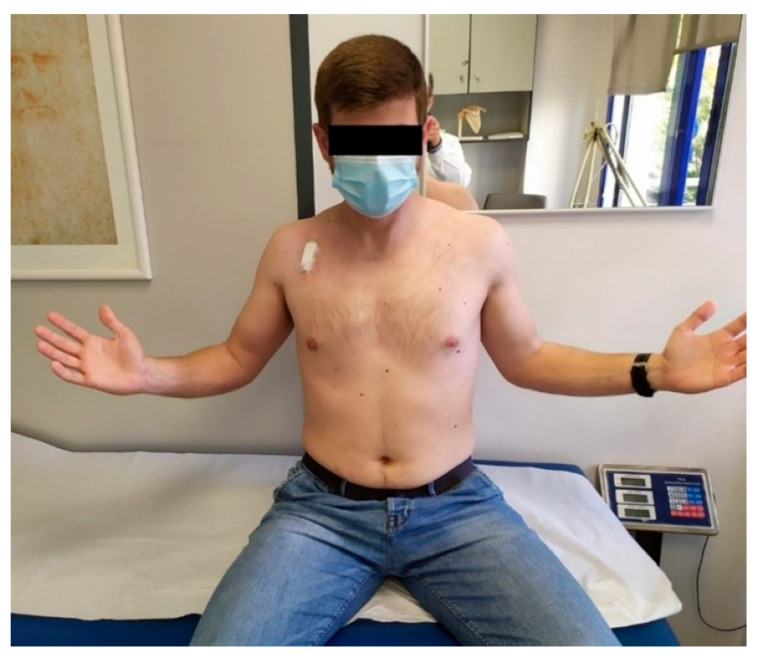
External rotation 10 days postoperatively.

**Figure 3:**
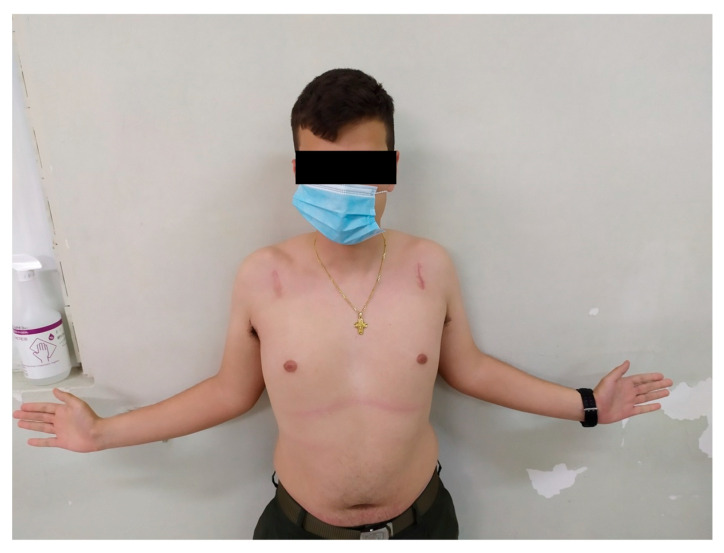
External rotation 8 weeks postoperatively.

The mean ER was 60° ± 5°, 75° ± 4°, and 80° ± 3° at 4, 8, and 12 weeks, respectively. Compared to the contralateral side, no external rotation deficit was observed post-operatively (Figure 2 and Figure 3).

**Figure 4:**
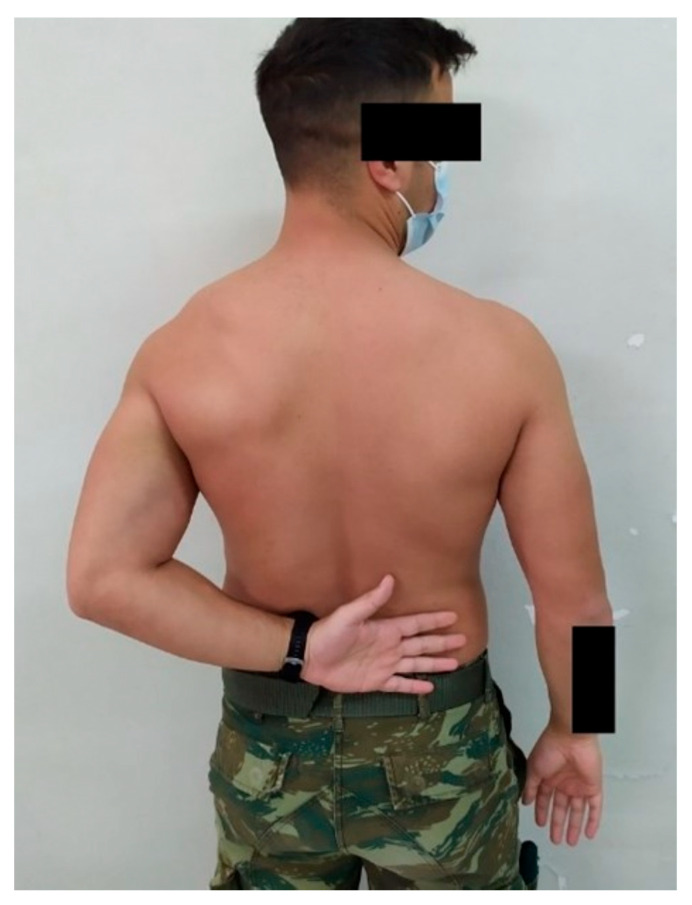
Internal rotation 4 weeks postoperatively.

**Figure 5:**
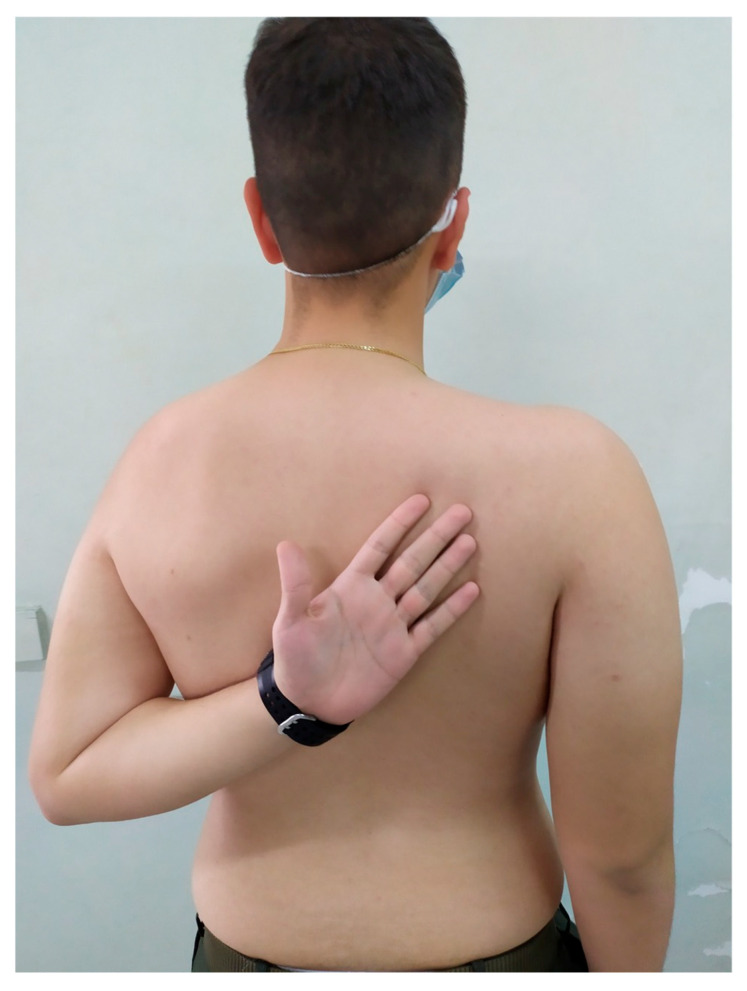
Internal rotation 8 weeks postoperatively.

The mean IR level was L3, T7, and T7 at 4, 8, and 12 weeks, respectively (Figure 4 and Figure 5). No complications or recurrent instability were recorded.

No significant side-to-side differences in ROM were observed at the final follow-up, and all patients achieved symmetrical function compared to the unaffected shoulder.

### 3.3. Complications and Satisfaction

Despite the fast, active, assisted self-rehabilitation protocol, no postoperative complications, graft displacements, infections, or recurrent instability events were recorded during follow-up. All grafts demonstrated satisfactory healing and positioning on radiographic evaluation at 12 weeks.

Patient-reported satisfaction was exceptionally high, with all individuals rating 9.5 ± 0.4 out of 10 at the 12-week mark. All patients returned to unrestricted daily activities, and most resumed military physical training by three months postoperatively.

## 4. Discussion

The findings of this study demonstrate that a structured self-rehabilitation protocol after a mini-open Latarjet procedure can achieve favorable short-term outcomes in young, motivated military patients. All participants regained a full range of motion by 12 weeks, experienced minimal postoperative pain, and reported high satisfaction, without complications or recurrent instability. These results suggest that, under appropriate guidance, self-directed rehabilitation can be a safe and effective alternative when access to supervised physiotherapy is limited.

Mini-open Latarjet is a safe and reliable procedure that has been shown to consistently restore glenohumeral stability when used to treat instability-related glenoid soft-tissue and osseous pathology in both cadaveric biomechanical studies and clinical outcome studies [2]. Patients with glenoid bone loss and contact athletes benefit more from the Latarjet procedure as they have lower failure rates and return to physical activities earlier than those with an anatomical Bankart repair [3,4,5,6,7,8,9]. Bankart repairs have higher complication, dislocation, and subluxation rates according to the literature [22,23]. There is also no need for the surgeon performing a mini-open Latarjet procedure to have arthroscopic proficiency. Other advantages of the technique are that there is adequate exposure to coracoid and glenoid defects, the avoidance of coracoclavicular ligaments and neurovascular structures damage during the osteotomy of the coracoid process, and there is no need for allografts, which decreases costs and heightens the potential for bony union with the native bone [24].

Since all our patients were army personnel, an early return to activities was crucial regarding patient satisfaction rates. All patients used a sling for the first three weeks after surgery. However, they were encouraged to perform gentle passive, active, and active-assisted shoulder range-of-motion exercises on their own due to COVID-19 pandemic restrictions in Greece. The self-rehabilitation protocol we proposed was concrete and straightforward, and all patients followed it without any issues. The remote follow-up component of our protocol aligns with growing evidence supporting the effectiveness of tele-rehabilitation in orthopedic postoperative care. Several studies have demonstrated that virtual supervision and home-based digital rehabilitation can achieve comparable functional outcomes to conventional in-person physiotherapy after shoulder procedures, while improving accessibility and reducing costs [3,15,16,17,18,19]. Although patients demonstrated high satisfaction and good clinical progress, our assessment of compliance relied on virtual follow-up discussions and patient self-reports. Objective adherence tracking was not feasible during the pandemic, and therefore, conclusions regarding compliance should be interpreted cautiously. While the positive outcomes observed in this cohort suggest that a structured self-rehabilitation protocol may be a feasible option when supervised physiotherapy is unavailable, the absence of a control group limits the ability to draw definitive comparative conclusions.

Nevertheless, several limitations must be acknowledged. First, the sample size was relatively small (18 patients) and all participants were drawn from a single military hospital, which may limit generalizability to broader populations. Second, the absence of a control group receiving standard supervised physiotherapy prevents direct comparison between approaches. Third, the study assessed only short-term outcomes up to 12 weeks; long-term follow-up would be necessary to evaluate recurrence rates, graft remodeling, and return-to-sport performance. Lastly, our cohort comprised exclusively male, young, fit, and disciplined military personnel, a population likely to adhere strictly to rehabilitation instructions. Outcomes may differ in older patients, women, or those with comorbidities.

Further research should focus on validating these findings through larger, multicenter studies with longer follow-up periods to assess long-term joint stability, graft healing, and return-to-duty or sports rates. Randomized controlled trials comparing structured self-rehabilitation with conventional supervised physiotherapy would clarify whether self-directed protocols can be broadly applied beyond highly motivated populations. Additionally, integrating digital tools such as smartphone-based exercise monitoring and telemedicine consultations could enhance patient adherence, enable real-time feedback, and further optimize outcomes in remote or resource-limited environments.

## 5. Conclusions

This study demonstrates that immediate self-rehabilitation after a mini-open Latarjet procedure is safe and effective in motivated military patients, without complications or mobility restrictions. Substantial early recovery and improvement in patients’ post-operative quality of life were observed in all patients at 12 weeks. These findings are particularly relevant in situations where access to physiotherapy is limited, such as during pandemics, deployments, or resource-constrained settings. A structured self-rehabilitation approach, when combined with appropriate patient education and remote follow-up, may represent a practical adjunct to standard postoperative care. Long-term follow-up and controlled comparative studies are required to determine whether these early improvements translate into durable functional and stability outcomes.

## Figures and Tables

**Table 1:** Clinical outcomes after mini-open Latarjet with self-rehabilitation. (Values expressed as mean ± SD where applicable.)

Outcome	Week 1	Week 2	Week 4	Week 8	Week 12
VAS (0–10)	1.2 ± 0.6	0.8 ± 0.5	0	0	0
Active Forward Flexion (°)	155 ± 10	170 ± 5	180	180	180
External Rotation (°)	-	-	60 ± 5	75 ± 4	80 ± 3
Internal Rotation Level	-	-	L3	T7	T7
Complications	None	None	None	None	None
Satisfaction (0–10)	-	-	-	-	9.5 ± 0.4

## Data Availability

The datasets used and analyzed during the current study are available from the corresponding author upon reasonable request.
